# Success Through Simplicity: What Other Artificial Intelligence Applications in Medicine Should Learn from History and ChatGPT

**DOI:** 10.1007/s10439-023-03287-x

**Published:** 2023-06-18

**Authors:** Sam Sedaghat

**Affiliations:** Department of Diagnostic and Interventional Radiology, University Hospital of Heidelberg, Heidelberg, Germany

**Keywords:** Artificial intelligence, AI, ChatGPT, Application, Future, Medicine

## Abstract

Many artificial intelligence (AI) algorithms have been developed for medical practice, but few have led to clinically used products. The recent hype of ChatGPT shows us that simple, user-friendly interfaces are one major factor in the applications’ popularity. The majority of AI-based applications in clinical practice are still far from simple-to-use applications with user-friendly interfaces. Therefore, simplifying operations is one key to AI-based medical applications’ success.

## Introduction

Many AI algorithms have been developed for medicine, but only a few have led to clinically used products and tools [1, 2]. The majority of AI-based applications in medicine are still far from simple-to-use applications with user-friendly operational interfaces. Besides the many challenges with using AI-based applications in medicine, such as privacy and data security, medical ethics, and risks of system failures, another issue hindering the broad use of AI-based applications in medical practice is the often-missing simplicity of operation in AI-based applications.

## Simplicity as a Key to Success in History

The first drive of Karl Benz in his Motorwagen on Ring Street of Mannheim (Germany) in 1886 counts as the first drive of an engine-powered car on public roads after years of innovation [3]. In the year 1908, the Ford Motor Company presented its Model T. The notion of Henry Ford’s car was to offer an “affordable, simple to operate, and durable vehicle” [4]. The Model T was one of the first mass-production vehicles [4], and Henry Ford knew in 1908 that simplicity of operation would be a major key to mass production.

When the Xerox Palo Alto Research Center designed an experimental personal computer to investigate how small and cost-effective a personal computer could be, no one would have thought of the mass use of computers and other computer-related applications decades later [5]. Further developments in the field of computer science are well known. Companies like Microsoft and Apple formed the future of the technological age today [6]. No one can imagine a world without personal computers, laptops, and smartphones. One of the major keys to the success of these systems and their applications is the simplicity of operation, consecutively making them simple to operate throughout all ages. Those applications usually react on one- or two-click commands and programming skills are generally unnecessary.

## ChatGPT’s Success Shows the Direction

ChatGPT (OpenAI, San Francisco, CA, USA) is an AI chatbot introduced recently [7]. Almost immediately after its release, people worldwide started using ChatGPT, leading to a continuous increase in the popularity of this application. The chatbot can be used in various ways, such as text summarization or telling creative stories [8]. ChatGPT has been tested for many purposes, such as educational purposes or academic writing [9]. Despite its enormous potential and sophisticated technology, one key factor that makes ChatGPT so popular is the simplicity of operation. ChatGPT gained broad popularity because it is an easy-to-use application that allows people of all ages to use it without sophisticated programming skills. Against this, if ChatGPT was a difficult-to-handle application, only a few people would use it today. It is worth looking back to Henry Ford’s notion of 1908 at this point, and what was true then is still true today: simplicity of operation is a major key to the success of new technologies! We learn from this that any AI-based application could fail if it is too difficult to operate for most people. AI applications must be reliable one- or two-click tools like ChatGPT, which do not require programming or more profound AI skills by the end user. Therefore, developing and improving user-friendly operational interfaces are essential and will determine the future of AI-based applications in clinical practice.

## Conclusion

History and the present show us that simplicity of operation is one major key to the success of new technological applications. Improving the simplicity of operation would help accelerate the broad implementation of AI-based applications in clinical practice.
